# Development of a radiomics model to diagnose pheochromocytoma preoperatively: a multicenter study with prospective validation

**DOI:** 10.1186/s12967-022-03233-w

**Published:** 2022-01-15

**Authors:** Jianqiu Kong, Junjiong Zheng, Jieying Wu, Shaoxu Wu, Jinhua Cai, Xiayao Diao, Weibin Xie, Xiong Chen, Hao Yu, Lifang Huang, Hongpeng Fang, Xinxiang Fan, Haide Qin, Yong Li, Zhuo Wu, Jian Huang, Tianxin Lin

**Affiliations:** 1 Department of Urology, Sun Yat-Sen Memorial Hospital, Sun Yat-Sen University, 107 Yan Jiang West Road, Guangzhou, 510120 Guangdong People’s Republic of China; 2 Guangdong Provincial Key Laboratory of Malignant Tumor Epigenetics and Gene Regulation, Sun Yat-Sen Memorial Hospital, Sun Yat-Sen University, Guangzhou, 510120 Guangdong People’s Republic of China; 3 Department of Urology, The Third Affiliated Hospital, Sun Yat-Sen University, Guangzhou, 510630 Guangdong People’s Republic of China; 4 Department of Neurology, Sun Yat-Sen Memorial Hospital, Sun Yat-Sen University, Guangzhou, 510120 Guangdong People’s Republic of China; 5 Department of Radiology, Sun Yat-Sen Memorial Hospital, Sun Yat-Sen University, Guangzhou, 510120 People’s Republic of China; 6 State Key Laboratory of Oncology in South China, Guangzhou, 510120 Guangdong People’s Republic of China

**Keywords:** Radiomics, Magnetic resonance imaging, Pheochromocytoma, Prediction, Nomogram

## Abstract

**Background:**

Preoperative diagnosis of pheochromocytoma (PHEO) accurately impacts preoperative preparation and surgical outcome in PHEO patients. Highly reliable model to diagnose PHEO is lacking. We aimed to develop a magnetic resonance imaging (MRI)-based radiomic-clinical model to distinguish PHEO from adrenal lesions.

**Methods:**

In total, 305 patients with 309 adrenal lesions were included and divided into different sets. The least absolute shrinkage and selection operator (LASSO) regression model was used for data dimension reduction, feature selection, and radiomics signature building. In addition, a nomogram incorporating the obtained radiomics signature and selected clinical predictors was developed by using multivariable logistic regression analysis. The performance of the radiomic-clinical model was assessed with respect to its discrimination, calibration, and clinical usefulness.

**Results:**

Seven radiomics features were selected among the 1301 features obtained as they could differentiate PHEOs from other adrenal lesions in the training (area under the curve [AUC], 0.887), internal validation (AUC, 0.880), and external validation cohorts (AUC, 0.807). Predictors contained in the individualized prediction nomogram included the radiomics signature and symptom number (symptoms include headache, palpitation, and diaphoresis). The training set yielded an AUC of 0.893 for the nomogram, which was confirmed in the internal and external validation sets with AUCs of 0.906 and 0.844, respectively. Decision curve analyses indicated the nomogram was clinically useful. In addition, 25 patients with 25 lesions were recruited for prospective validation, which yielded an AUC of 0.917 for the nomogram.

**Conclusion:**

We propose a radiomic-based nomogram incorporating clinically useful signatures as an easy-to-use, predictive and individualized tool for PHEO diagnosis.

**Supplementary Information:**

The online version contains supplementary material available at 10.1186/s12967-022-03233-w.

## Introduction

Pheochromocytoma (PHEO) is a rare neuroendocrine catecholamine-secreting tumor originating from the chromaffin cells, with an annual incidence of approximately 1–2/100,000 person-years [1, 2]. Predicting PHEO prior to surgery can alleviate perioperative mobility and mortality as they can produce excessive catecholamine if perioperatively improperly handled; leading to consequential life-threatening hypertension, arrhythmia, and stroke [3]. Therefore, preoperatively discriminating PHEOs from other adrenal tumors is crucial for appropriate treatment planning.

Abdominal/pelvic multiphasic computed tomography (CT) or magnetic resonance imaging (MRI) is commonly used in cancer diagnosis [4] and has been recommended as the most common non-invasive modality to diagnose PHEO [5]. MRI is a morphologic imaging procedure that can differentiate PHEOs from other adrenal tumors such as adenomas and metastases [6, 7] since PHEOs are generally hyperintense on T2-weighted images [8]. However, due to atypical signs on T2-weighted MR images, about 35% of PHEOs can be misclassified [9, 10]. In addition, lesions of PHEOs with hemorrhage and necrosis can be heterogeneous [8]. PHEOs may also mimic other adrenal masses on the traditional radiological analysis due to the overlap in imaging features and non-specific clinical findings [11–13]; encapsulating the notion of “imaging chameleon” [14]. Therefore, diagnosing PHEO accurately and timely remains a challenge [14–16]. As a result, developing a more accurate preoperative imaging tool to diagnose PHEO is in urgent need.

Computational medical imaging, also called radiomics, involves the analysis and translation of medical images into quantitative data [17, 18]. Based on high-throughput imaging features, the minable data from radiomics can improve the diagnostic, prognostic, and predictive accuracy, bridging the gap between clinical imaging and personalized medicine [19, 20]. Furthermore, radiomics features have the advantages to evaluate a tumor and its microenvironment, characterization of spatial heterogeneity, and longitudinal evaluation of cancer evolution. There have been many applications of radiomics in cancer diagnosis and prediction such as rectal cancer, breast cancer, and bladder cancer [21–24]. In addition, Yi et al. developed a CT-based radiomics signature to differentiate subclinical pheochromocytoma from lipid-poor adenoma. Nevertheless, as a single-center study, the patient population in the study was relatively small and homogeneous. And the study only used a region of interest (ROI) of the adrenal lesions for radiomics analysis, making it unable to effectively reveal the heterogeneity of the entire lesion. Further studies are warranted due to limitations. However, to the best of our knowledge, there is no publication evaluating whether an MRI-based radiomics signature would facilitate the preoperative diagnosis of PHEO.

Thence, the purpose of this study was to investigate whether an MRI-based radiomics analytics was capable of preoperative differentiation of PHEOs and non-PHEOs (e.g. benign adrenocortical adenomas, adrenocortical carcinomas, other pathologies). In addition, a radiomic-clinical model was developed and then validated in an internal validation set, an external validation set, and a prospective validation set.

## Methods and materials

### Patients

This study was approved by the institutional review board at the Sun Yat-sen Memorial Hospital of Sun Yat-sen University and the Third Affiliated Hospital of Sun Yat-sen University (Guangzhou, China). Written informed consent was obtained from each patient. The retrospective cohort of this study comprised of an evaluation of the institutional database for medical records from September 2010 to May 2019 to identify patients with adrenal mass who underwent surgical resection with curative intent. Patients for the prospective validation were treated at our institute between June 2019 and June 2020 (ClinicalTrials.gov identifier: ChiCTR1900028520).

A total of 305 patients were included in this cohort study based on the following criteria. The inclusion criteria for patient selection were: (i) underwent adrenalectomy; (ii) pathologically confirmed as adrenal tumor; (iii) had preoperative MRI examination. The exclusion criteria were: (i) poor imaging quality or imaging artifacts; (ii) pathologically confirmed diagnosis of adrenal hyperplasia, adrenal cyst, or adrenal angiolipoma. The non-PHEO was defined as adrenal tumors other than pheochromocytoma, adrenal hyperplasia, adrenal cyst, and adrenal angiolipoma. Then, 239 patients treated in the Sun Yat-sen Memorial Hospital were divided into a training cohort (N = 166 patients with n = 170 lesions diagnosed between September 2010 and October 2013) and an internal validation cohort (N = 73 patients with n = 73 lesions diagnosed between November 2013 and May 2019) in a ratio of 7:3. Patients treated in the Third Affiliated Hospital of Sun Yat-sen University were used as the external validation set (N = 66 patients with n = 66 lesions diagnosed between August 2010 and May 2019). Finally, 25 patients with 25 adrenal lesions were recruited to prospectively validate the model.

**Fig. 1:**
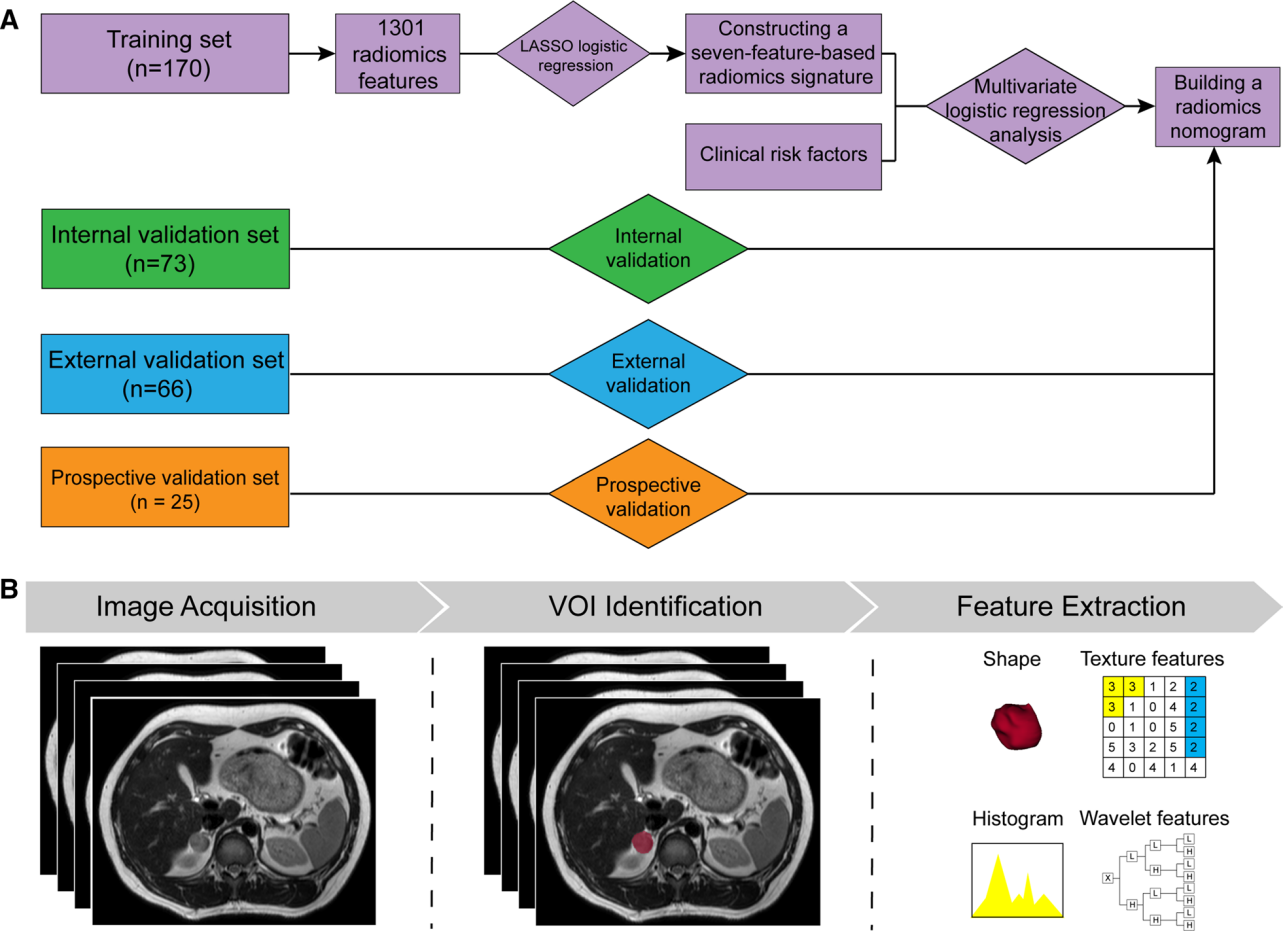
The radiomics workflow and study flowchart. VOI, volume of interest

The clinical and pathological data retrieved from the medical records included sex, age, tumor location, smoking history, presence of hypertension, and symptom number (symptoms include headache, palpitation, and diaphoresis). Pathological examination of adrenal lesions was reviewed by two pathologists with more than 10 years of experience. Any disagreement was resolved by consensus. Since some patients had bilateral lesions, each lesion was considered as a subject to be measured in this study. The entire study flowchart is presented in Fig. 1A and the recruitment pathway is shown in Additional file 1: Fig. S1.

### MRI image acquisition, segmentation, and feature extraction

Figure 1B presents the radiomics procedure. Axial T2-weighted images were used for radiomics analyses, performed using a 3D Slicer (version 4.9.0, https://www.slicer.org), an open-source software program widely used for image visualization and segmentation [25]. The Additional file 1: Supplementary Methods describes in detail the MRI image acquisition, segmentation, and the algorithms for radiomics feature extraction along with their reproducibility.

### Radiomics signature construction and performance assessment

The least absolute shrinkage and selection operator (LASSO) logistic regression algorithm, which is suitable for the regression of high-dimensional data [26], was used to select the most impactful predictive features in the training set. Then, a radiomics signature was constructed and the radiomics score was calculated for each patient via a linear combination of the selected features that were weighted by their respective LASSO coefficients.

The potential use of the radiomics signature to diagnose PHEO was first assessed in the training set and validated in the internal and external validation sets by using a Mann–Whitney *U* test. Then, stratified analyses were performed (Additional file 1: Data Supplement). Furthermore, discrimination of the radiomics signature was assessed using the receiver operating characteristic (ROC) curve and the area under the ROC curve (AUC). An optimism-corrected AUC was also calculated by bootstrapping method (2000 bootstrap resamples) to obtain stable optimism-corrected estimates [27].

### Development of a radiomic-clinical nomogram and performance assessment

Multivariable logistic regression analysis began with the radiomics signature and the clinical candidate predictors in the training set. The backward stepwise selection was applied using the Akaike’s Information Criterion (AIC) as the stopping rule [28]. Based on the result of the multivariable logistic analyses, a radiomic-clinical nomogram was built. Meanwhile, variance inflation factors (VIFs) were calculated for evaluating the multicollinearity among variables in the regression model. The logistic regression formula for calculating the risk score was also presented.

The discrimination and calibration of the radiomic-clinical nomogram were evaluated in the training set. AUC and optimism-corrected AUC were employed to evaluate the discrimination of the radiomic-clinical nomogram, and calibration curve was used to assess the calibration, accompanied by the Hosmer–Lemeshow test.

### Internal and external validation of the radiomic-clinical nomogram

The logistic regression formula formed in the training set was applied to all patients of the internal and external validation sets. Meanwhile, the performances of the nomogram were evaluated in the internal or external validation set using the AUC calculation and calibration analysis.

### Models comparison and clinical usefulness evaluation

To further evaluate the radiomic-clinical nomogram applicability, we compared the radiomic-clinical model to a clinical model incorporating clinical independent predictors alone, which was identified by a multivariate logistic regression analysis. The ROC curves were plotted and AUCs were calculated to quantify the discriminative ability of each model.

Decision curve analysis (DCA) was conducted to determine the clinical usefulness of the radiomic-clinical nomogram by quantifying the net benefits at different threshold probabilities [29], and the clinical model was also compared.

### Prospective validation of the radiomics model

Following the construction of the radiomic-clinical model, 25 patients with adrenal tumors were enrolled and used as prospective validation. In the prospective validation set, ROC analyses were used to evaluate the performance. In addition, the associated classification measures, including sensitivity, specificity, positive predictive value, and negative predictive value were also calculated for the radiomic-clinical nomogram.

### Statistical analyses

All statistical analyses were performed using the R software, version 3.5.3 (https://www.r-project.org/). The LASSO logistic regression was performed using the “glmnet” package. The nomogram and calibration curve were plotted using the “rms” package. Hosmer–Lemeshow test was performed using the “vcdExtra” package, and VIF was calculated using the “car” package. DCA was performed using the “dca.r” function. Statistical significance was two-sided, with significance level at *P* < 0.05.

## Results

### Patients characteristics

The detailed clinicopathological characteristics of the patients in the training (N = 166), internal validation (N = 73) and external validation (N = 66) sets are summarized in Table 1. Of the total 305 retrospective patients included in the study, 133 (43.6%) were men, and the median (interquartile range, IQR) age was 49.0 (39.0–57.0) years. Among them, 23.9% (74/309) of the lesions were diagnosed as PHEOs. The clinical data of the prospective validation set (N = 25) is provided in Additional file 1: Table S1.

**Table 1:** Baseline characteristics of the patients

	Training set (*N* = 166)	Internal validation set (*N* = 73)	External validation set (*N* = 66)
Sex			
Male	67 (40.4%)	32 (43.8%)	34 (51.5%)
Female	99 (59.6%)	41 (56.2%)	32 (48.5%)
Age, years			
Median (Interquartile range)	49.0 (39.0–57.0)	47.0 (37.0–55.0)	49.0 (36.0–57.0)
Symptom number*			
0	117 (70.5%)	46 (63.0%)	47 (71.2%)
1	32 (19.3%)	17 (23.3%)	14 (21.2%)
2	10 (6.0%)	7 (9.6%)	3 (4.5%)
3	7 (4.2%)	3 (4.1%)	2 (3.1%)
Hypertension			
Yes	80 (48.2%)	30 (41.1%)	44 (66.7%)
No	86 (51.8%)	43 (58.9%)	22 (23.3%)
Smoker			
Yes	22 (13.3%)	12 (16.4%)	10 (15.2%)
No	144 (86.7%)	61 (83.6%)	56 (84.8%)
Tumor location			
Left	89 (52.4%)	35 (47.9%)	34 (51.5%)
Right	81 (47.6%)	38 (52.1%)	32 (48.5%)
MRI-determined tumor size, cm**			
Median (Interquartile range)	3.3 (2.0–4.6)	3.2 (2.2–5.0)	2.4 (1.7–4.6)

MRI: magnetic resonance imaging

*Symptoms include headache, palpitation, and diaphoresis

**Each individual lesion was regarded as a subject to be measured in these variables (There were n = 170 lesions in the training set, n = 73 lesions in the internal validation set and n = 66 lesions in the external validation set)

### Radiomics signature construction and performance assessment

**Fig. 2:**
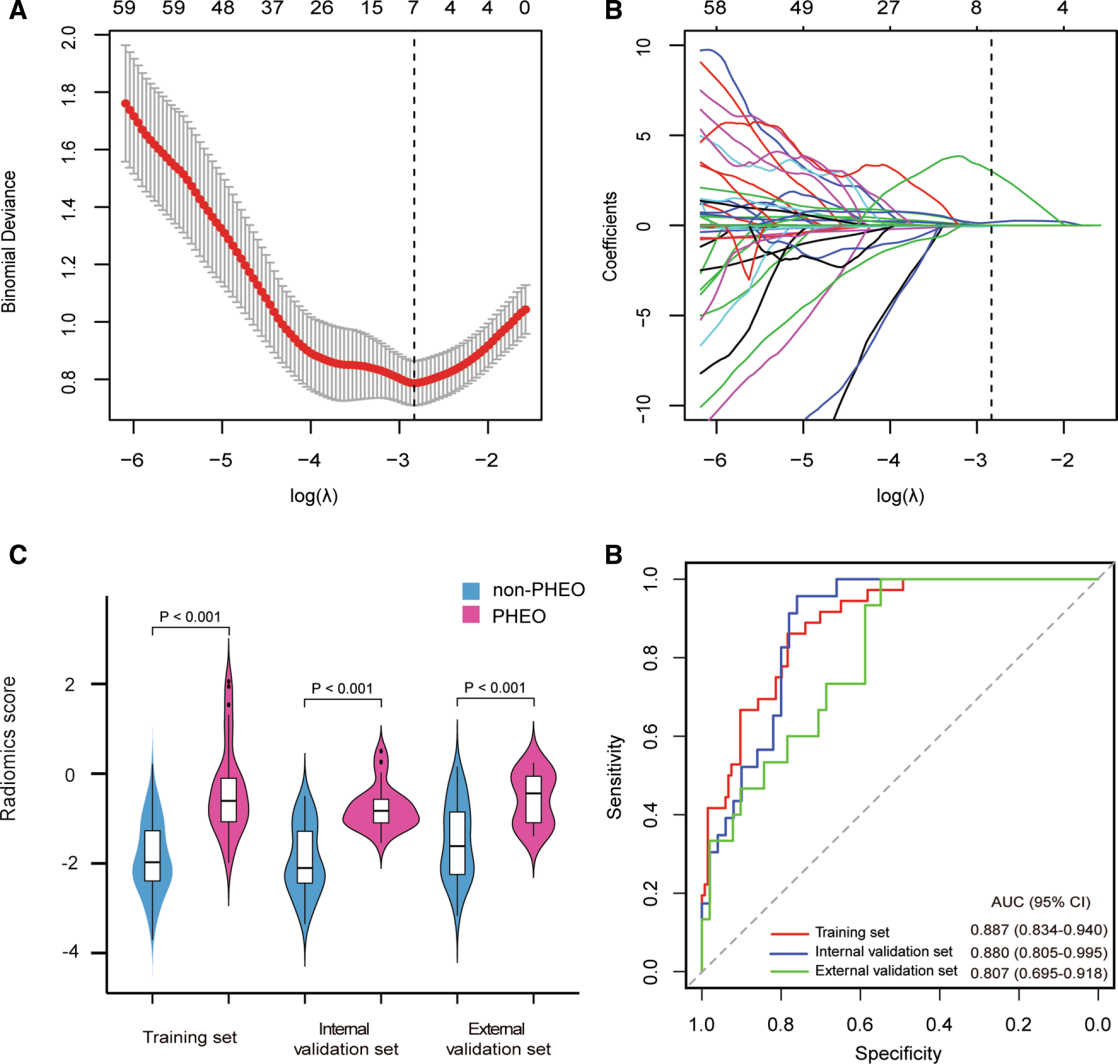
Development of the radiomics signature and performance assessment. **A** Selection of the tuning parameter (λ). The tuning parameter lambda (λ) was selected by the LASSO method based on tenfold cross-validation via minimum criteria. The binomial deviance was plotted versus the log-transformed λ. Based on the minimum criteria, the calculated optimal values were plotted as the dotted vertical line. The optimal λ value of 0.059 with log (λ) of − 2.833 was selected. **B** LASSO coefficient profiles of the 1301 radiomics features. Seven stable features with nonzero coefficients were selected, according to the vertical line plotted at the optimal λ value. **C** Boxplots of the radiomics score in the training, internal and external validation sets. **D** ROC curves of the radiomics signature in the training, internal and external validation sets

In total, 1301 radiomics features were extracted from each lesion. Among them, 7 features were selected as potential predictors using the LASSO logistic regression algorithm in the training set (Fig. 2A and B). The selected features and their corresponding coefficients are shown in the Supplementary Methods.

The radiomics scores between the PHEO and non-PHEO patients demonstrated significant difference among the training set (median [interquartile range], − 0.608 [− 1.073 to − 0.102] vs. − 1.977 [− 2.394 to − 1.274], respectively, *P* < 0.001, Fig. 2C), the internal validation set (median [interquartile range], − 0.829 [− 1.099 to − 0.572] vs. − 2.103 [− 2.440 to − 1.283], respectively, *P* < 0.001, Fig. 2C) and the external validation set (median [interquartile range], − 0.439 [− 1.097 to − 0.058] vs. − 1.616 [− 2.249 to − 0.854], respectively, *P* < 0.001, Fig. 2C). Furthermore, significant association of the radiomics score between the PHEO and non-PHEO patients was also found in the stratified analysis (Additional file 1: Table S2). Further, the radiomics signature yielded an AUC of 0.887 (95% CI 0.834–0.940) and an optimism-corrected AUC of 0.886 in the training set, which was validated in the internal and external validation sets with AUCs of 0.880 (95% CI 0.805–0.995) and 0.807 (95% CI 0.695–0.918), respectively (Fig. 2D).

### Radiomic-clinical nomogram construction and performance assessment

**Fig. 3:**
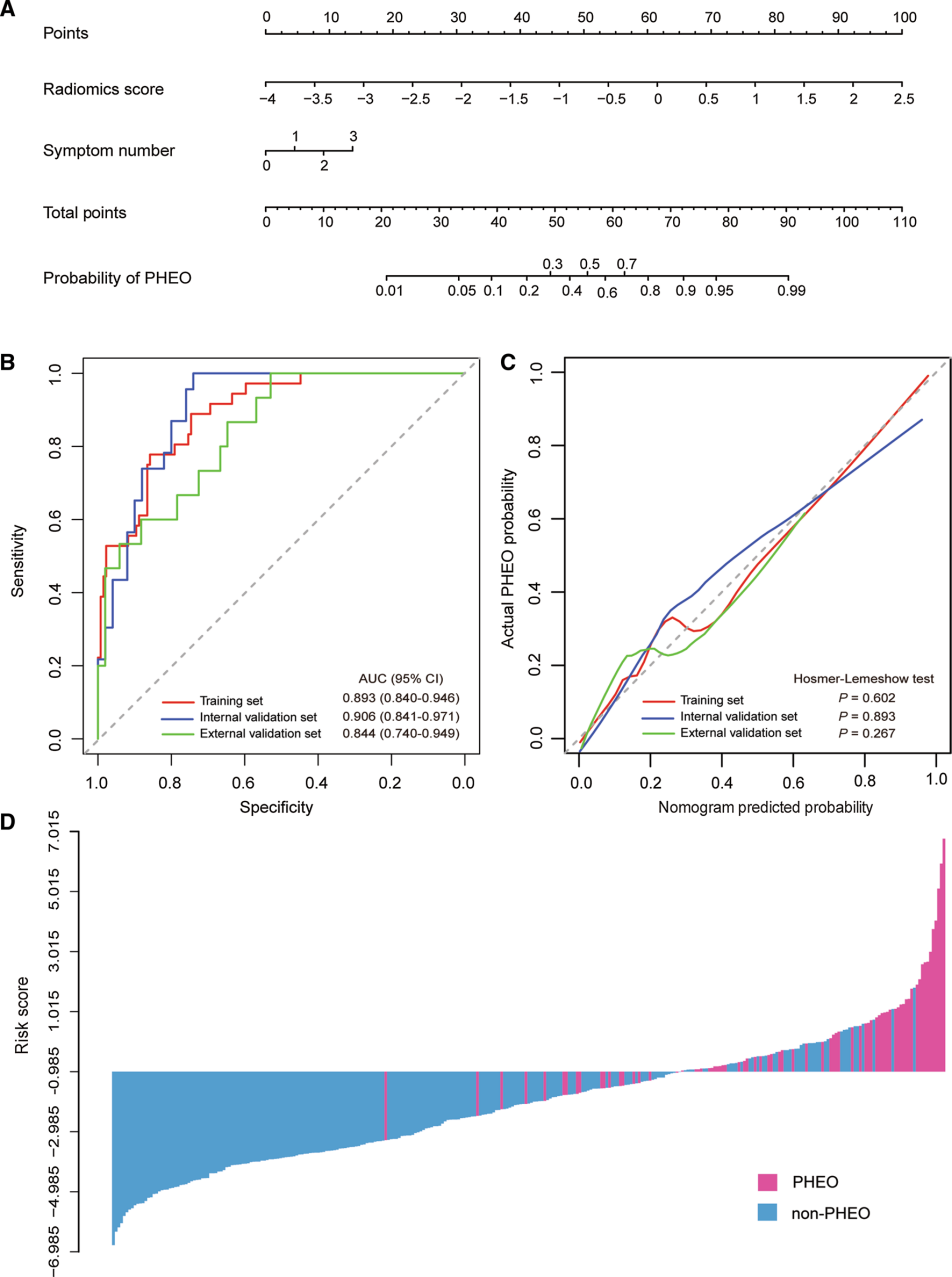
The radiomic-clinical nomogram and its performance. **A** The radiomic-clinical nomogram was developed to distinguish PHEOs from other adrenal lesions. **B** ROC curves of the radiomic-clinical nomogram in the training, internal and external validation sets. **C** Calibration curves of the nomogram in the training, internal and external validation sets. The calibration curve presents how well the predicted probabilities agree with the observed probabilities. The diagonal dotted line indicates the ideal prediction by the ideal model. The solid lines present the prediction value of the nomogram. A closer fit of the solid line to the diagonal dotted line demonstrates a better prediction. **D** The calculated risk scores for each patient within the combined training, internal and external validation datasets

The radiomics signature and symptom number were identified as independent predictors to distinguish PHEO from other adrenal lesions. For the collinearity diagnosis, all VIFs were less than 4 (ranging from 1.072 to 1.451), which indicated that there was no collinearity. To ensure easy use of the predictive model, we presented it as a nomogram (Fig. 3A). The logistic regression formula for calculating the risk score was as follows: (2.035 × radiomics score) + (0.602 × symptom number) + 0.781.

The radiomic-clinical nomogram achieved good discrimination with an AUC of 0.893 (95% CI 0.840–0.946) in the training set (Fig. 3B). The optimism-corrected AUC of the nomogram was 0.892. In addition, an optimal risk score cutoff value of − 0.985 was defined according to the maximum Youden index. The calibration curve demonstrated good agreement between predicted and observed probabilities in the training set (Fig. 3C). The Hosmer–Lemeshow test yielded a nonsignificant statistic (*P* = 0.602), indicating that there was no departure from a perfect fit.

### Internal and external validation of the radiomic-clinical nomogram

The radiomic-clinical nomogram yielded a favorable AUC of 0.906 (95% CI 0.841–0.971, Fig. 3B) in the internal validations set and yielded an AUC of 0.844 (95% CI 0.740–0.949, Fig. 3B) in the external validation set. Good calibration was also observed both in the internal and external validation sets along with nonsignificant statistics (*P* = 0.893 and *P* = 0.267, respectively) in the Hosmer–Lemeshow tests (Fig. 3C).

The waterfall plot showed the distribution of risk scores and pathologic diagnosis for all lesions (Fig. 3D). The specificity, sensitivity, and accuracy of the radiomic-clinical model detection of PHEO were 0.750, 0.857, and 0.840 in the internal validations set.

### Model comparison and clinical usefulness evaluation

**Fig. 4:**
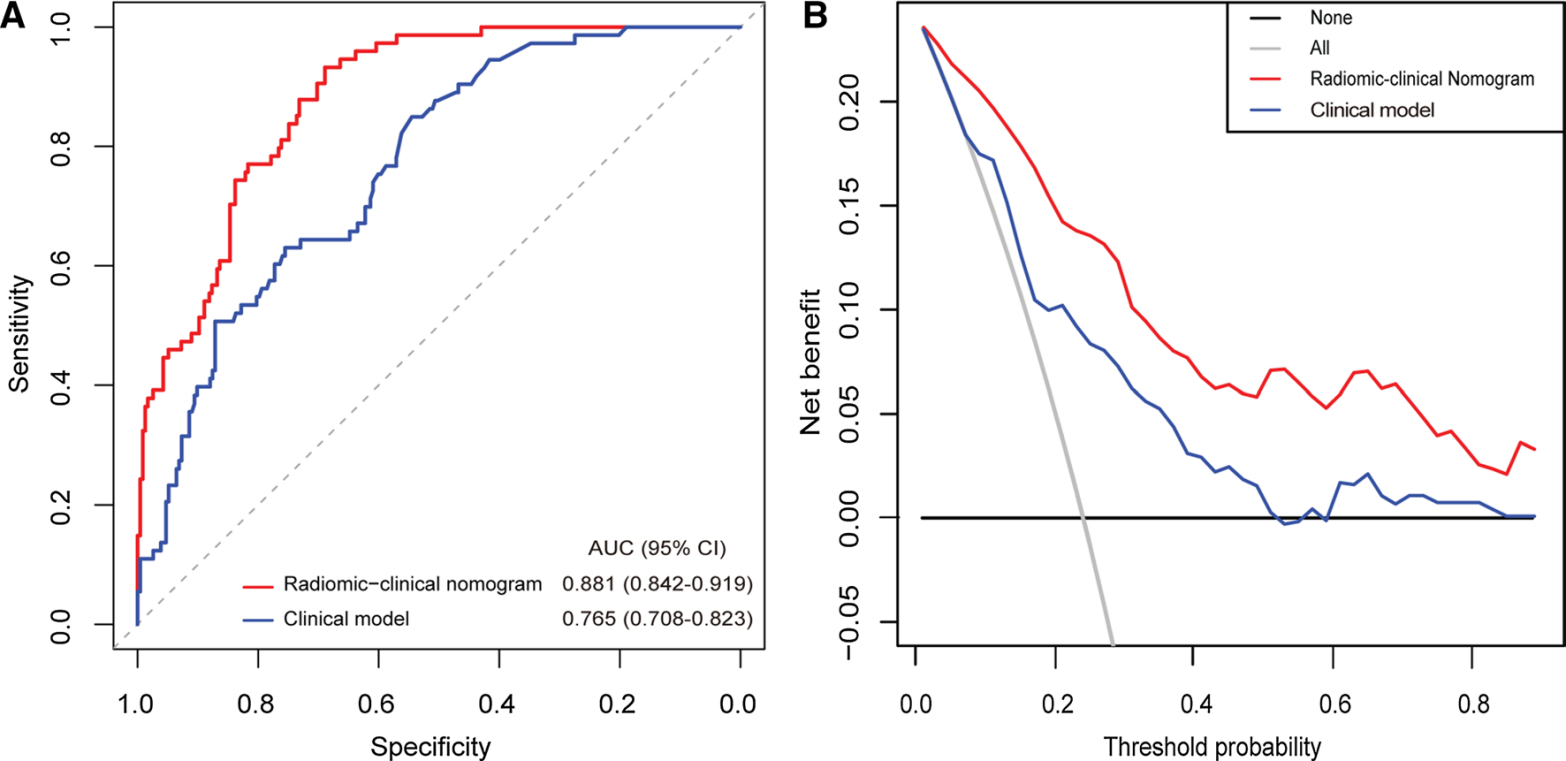
Receiver operating characteristic analysis (**A**) and decision curve analysis (**B**) of the radiomics-clinical model and clinical model in the combined training, internal and external validation sets

In the training set, we further analyzed the eight clinical candidate variables using multivariate logistic regression to construct the clinical model. As a result, the clinical model incorporates two predictors, i.e., symptom number and MRI-determined tumor size (Table 2). In the combined training, internal and external validation sets, the radiomic-clinical nomogram achieved significantly higher AUC than the clinical model (AUC [95% CI], 0.881 [0.842 to 0.919] vs*.* 0.765 [0.708 to 0.823], respectively, *P* < 0.001, Fig. 4A). The DCA showed that using the radiomic-clinical nomogram to predict PHEO added more net benefit than the clinical model (Fig. 4B). Similar findings were found in both the training and the validation sets (Additional file 1: Fig. S2).

**Table 2:** Multivariate logistic regression analysis of the radiomics score and clinical candidate predictors in the training set

Variables and intercept	Univariate model			Radiomic-clinical multivariate model			Clinical multivariate model		
	β	OR (95% CI)	*P*	β	OR (95% CI)	*P*	β	OR (95% CI)	*P*
The radiomics score (per 0.1 increase)	0.207	1.230 (1.152 to 1.336)	< 0.001^*^	0.204	1.226 (1.146 to 1.332)	< 0.001^*^	–	–	–
Sex (male vs. female)	0.423	1.526 (0.314 to 7.982)	0.283	–	–	–	–		
Age, years (continuous)	0.004	1.004 (0.978 to 1.032)	0.759	–	–	–	–	–	–
Symptom number	0.824	2.279 (1.487 to 3.594)	< 0.001^*^	0.602	1.826 (1.013 to 3.280)	0.042^*^	0.852	2.344 (1.511 to 3.731)	< 0.001^*^
Hypertension (no vs. yes)	0.201	1.222 (0.585 to 2.574)	0.593	–	–	–	–	–	–
Smoker (no vs. yes)	− 0.597	0.550 (0.124 to 1.742)	0.359	–	–	–	–	–	–
Tumor location (left vs. right)	0.692	1.998 (0.951 to 4.317)	0.071	–	–	–	–	–	–
MRI-determined tumor size, cm (continuous)	0.173	1.189 (1.056 to 1.346)	0.005^*^	–	–	–	0.182	1.200 (1.060 to 1.365)	0.004^*^
Hyperintense on a T2 weighted MRI (no vs. yes)	2.014	7.495 (2.139 to 47.541)	0.007^*^	–	–	–	–	–	–
Intercept	–	–	–	0.781			− 2.586	–	–

CI: confidence interval; MRI: magnetic resonance imaging; OR: odds ratio

*P < 0.05

### Prospective validation of the radiomics model

Conspicuously, the radiomics signature predicted PHEO in the prospective validation set yielded an AUC of 0.881 (95% CI 0.726 to 1.000, Additional file 1: Fig. S3A), and the radiomics scores showed a significant difference between the PHEO and non-PHEO patients (median [interquartile range], − 0.246 [− 1.061 to 1.006] vs. − 2.466 [− 3.061 to − 1.433], respectively, *P* = 0.020, Additional file 1: Fig. S3B). In addition, the radiomic-clinical model performed well in the prospective validation for prediction of the PHEO, with an AUC of 0.917 (95% CI 0.801 to 1.000, Additional file 1: Fig. S3A). These findings suggest the ability of the model to identify the PHEO.

## Discussion

In this study, we used high-throughput extraction of data-characterization algorithms to extract radiomics features and constructed a nomogram with combined radiomics features and clinical risk factors to distinguish PHEOs from other adrenal lesions [30, 31]. The nomogram was validated using different validation sets and demonstrated promising reproducibility and reliability of the prediction model for potential clinical usage in the pre-operative diagnosis of PHEOs.

As we have known, PHEO was notoriously difficult to diagnose as a rare neuroendocrine tumor causing a myriad of clinical symptoms. PHEOs are heterogeneous at imaging, including cystic change, hemorrhage, calcification, intracellular lipid, and malignancy [32]. Most show high morbidity and mortality due to excessive catecholamine production, leading to hypertension, arrhythmia, and stroke [33]. It would be lethal in uncontrolled and unrecognized cases. The ESE/ENSAT guideline has suggested that determination of metanephrines might be eliminated in case of adrenal mass with an unenhanced CT attenuation value ≤ 10 HU [34]. However, this recommendation was based on a small sample size of patients with an unenhanced attenuation value > 10 HU [35, 36]. The specificity of the biochemical tests for PHEOs depends quite mainly on preanalytical criteria which maintains the use of some affected drugs (e.g. dopamine D2 receptor antagonists) and the need of a correctly collected 24 h urine and blood sampling [37, 38]. And Edward’s study showed that the probability of pheochromocytoma in adrenal incidental tumors with unenhanced CT attenuation ≤ 10HU was low, which did not support the widespread clinical practice to determine metanephrines in every patient with adrenal lesions [39]. Furthermore, the determination of metanephrines is rather expensive. The rate of false-positive results might be increased because the optimal preanalytical conditions were difficult to create, which makes it do more diagnostic tests to rule out PHEO at higher costs [40].

Although previous studies have been reported that the overall diagnostic performance was good for diagnosing PHEOs, it should be noted that the sensitivity was relatively low (51.7–58.0%) with traditional imaging technology [39, 41, 42]. With Gallium-Dotatate PET CT pheochromocytomas and paragangliomas can be visualized with high sensitivity and in case of malignant pheochromocytomas metastases can be demonstrated [43]. Nonetheless, its application is limited due to the expensive inspection and the difficulty of popularization in primary hospitals. Apart from adrenal incidentoma, it is possible that the method might be useful in applications where MRI is specifically indicated for imaging of PHEO. This includes clinical contexts in which radiation exposure should be minimized (e.g., children, young women of child bearing age). Use of MRI might also be considered to minimize radiation exposure during periodic repeated imaging studies in patients with mutations of tumor-susceptibility genes.

Application of radiomics analysis has been recognized as an important technology [18, 44, 45]. The high-dimensional imaging features can acquire more detailed information about the tumor which cannot be detected easily by the naked eye. And different pathological types of tumors exhibit different values of radiomics features, which might be an underlying mechanism of applying radiomics in tumor classification [46]. Our study analyzed the images with 3D-VOI method, extracted a total of 1301 radiomic features and built a radiomic signature using seven selected radiomics features after reduction of redundant features. Some selected features describe the distribution of voxel intensity of the VOI region, such as ‘Mean’ and ‘Skewness’, while some selected texture features like ‘Entropy’ hold information about the spatial heterogeneity of the lesion. However, the interpretation of features remains a challenge in radiomic research, and further studies are warranted to explore the potential biological significance underlying the selected radiomics features. In our study, we did establish an MRI-based radiomics signature which demonstrated strong potential as promising indicators for the diagnosis of PHEOs to aid physicians to more accurately diagnose the PHEOs.

Furthermore, by incorporating clinical predictors, we developed and validated a radiomics nomogram in our study, which has the ability to diagnose the PHEOs. Odds ratio of symptom number is 1.826 in the multivariable logistic regression analysis, suggesting that the greater the symptom number is, the greater the probability of PHEO diagnosis (Table 2). Indeed, symptoms like palpitations, headache, and diaphoresis are significant when considering a possibility of PHEO diagnosis [34]. The model demonstrated high discriminatory power, with AUCs greater than 84.4%. As such, it could stratify patients with PHEOs and non-PHEOs. Patients with PHEO could receive preoperative medication for further surgical treatment or even targeted therapy including germline mutations in *SDHB* or fusions involving *MAML3* [33]. That is, it could be helpful for clinicians to determine personalized treatment strategies preoperatively. Thus, the radiomic-clinical model may further increase the accuracy of the PHEOs diagnosis and minimize the cases of misdiagnosis and missed diagnosis.

Intriguingly, when compared with the clinical model, the radiomic-clinical model was superior in both the training and validation sets. This suggests that the MRI imaging radiomics features are more representative of the tumors and the radiomic-clinical model is not only a simple combination of radiomics features but also a synergy between intratumor heterogeneity and clinical variables. The underlying explanation for the good performance of our radiomic model may be that the internal structure of lesion heterogeneity reflected by radiomic characteristics is related to the biological behavior and microstructure of adrenal tumor [47], which were critical factors influencing the efficacy in predicting PHEOs preoperatively.

To our knowledge, this is the first study to show that MRI-based radiomics can be used to distinguish PHEOs from other adrenal lesions. The pros of the findings of this study include: first, we used an open-sourced software for the radiomics procedure instead of custom-developed software, which makes it possible to further validate the model even conducted by other institutions [48]; second, the model developed using the retrospective data was not only validated using an internal validation set but was also further validated in the prospective set; third, the predictors, radiomics signature and symptom number, in the nomogram were feasibly obtained from routine MRI scans and medical history taking, thereby enhancing their clinical availability and usability.

However, there were several limitations to be acknowledged. First, it was mainly designed using two institutional retrospective studies with a modest number of patients that may not be representative in other institutions. Accordingly, multicenter prospective trials with larger patient samples are needed in order to improve clinical efficacy [49]. Second, semi-automatic tumor segmentation contained complex operation and possibly man-made interference. A more stable and time-saving method such as automatic segmentation could be applied to the radiomics analysis [50]. Third, our study did not involve nuclear imaging, which limits our further clinical outreach and application. Fourth, the data of biochemical tests for PHEOs were not included in our presented model due to the lack of these data in our retrospective study. Further studies are warranted to investigate whether the biochemical tests can improve the performance of the radiomics model. Future work should involve analyses of the dependencies between radiomics features and clinical variables or genetic changes, which may further improve the diagnostic model.

## Conclusions

In conclusion, we developed a novel radiomics model combining the radiomics signature and symptom number for predicting the presence of PHEOs, against other adrenal lesions, before initial treatment. It can be used as a noninvasive, safe, simple-to-implement, and accurate method in the daily setting, after wider prospective validation.

## Supplementary Information

**Additional file 1: Fig. S1.** Recruitment pathways. **Fig. S2.** Receiver operating characteristic analysis (**A**–**C**) and decision curve analysis (**D**–**F**) of radiomic-clinical nomogram model and clinical model in the training set, internal validation set, and external validation set, respectively. **Fig. S3.** Performance of the radiomics signature and radiomic-clinical model in the prospective validation set. (**A**) ROC curve of the radiomics signature in the prospective validation set. (**B**) Boxplots of the radiomics score in the prospective validation set. (**C**) ROC curve of the radiomic-clinical nomogram in the prospective validation set. **Table S1.** The clinical data of the prospective validation set. **Table S2.** Stratified analysis of the association between the radiomics signature and the pathological characteristics of adrenal lesions in the combined training, internal validation and external validation sets. **Table S3.** Stratified analysis of the association between the radiomics signature and MRI parameters in all enrolled patients.

## Data Availability

The datasets used during the current study are available from the corresponding author on reasonable request.
