# Supplementary material for: Development of a radiomics model to diagnose pheochromocytoma preoperatively: a multicenter study with prospective validation
Source: J Transl Med. 2022 Jan 15;20:31. doi: 10.1186/s12967-022-03233-w (PMC8760711; doi:10.1186/s12967-022-03233-w)
Supplement: Supplementary file 1 — Additional file 1: Fig. S1. Recruitment pathways. Fig. S2. Receiver operating characteristic analysis (A–C) and decision curve analysis (D–F) of radiomic-clinical nomogram model and clinical model in the training set, internal validation set, and external validation set, respectively. Fig. S3. Performance of the radiomics signature and radiomic-clinical model in the prospective validation set. (A) ROC curve of the radiomics signature in the prospective validation set. (B) Boxplots of the radiomics score in the prospective validation set. (C) ROC curve of the radiomic-clinical nomogram in the prospective validation set. Table S1. The clinical data of the prospective validation set. Table S2. Stratified analysis of the association between the radiomics signature and the pathological characteristics of adrenal lesions in the combined training, internal validation and external validation sets. Table S3. Stratified analysis of the association between the radiomics signature and MRI parameters in all enrolled patients. [file 12967_2022_3233_MOESM1_ESM.docx]

Supplementary Information

**Development of a Radiomics Model to Diagnose Pheochromocytoma Preoperatively: A Multicenter Study with Prospective Validation**

Jianqiu Kong^1,2^*, Junjiong Zheng^1,2^*, Jieyin Wu^3*^, Shaoxu Wu^1,2^, Jinhua Cai^4^, Xiayao Diao^1,2^, Weibin Xie^1,2^, Xiong Chen^1,2^, Hao Yu,^1,2^, Lifang Huang^1,2^, Hongpeng Fang^3^, XinXiang Fan^1,2^, Haide Qin^1,2,6^, Yong Li ^5^, Zhuo Wu^5^, Jian Huang ^1,2,6^†, Tianxin Lin^1,2,6^†

Contents

[SUPPLEMENTARY METHODS 3](#_Toc10470)

[Radiomics Procedure 3](#_Toc22127)

[Radiomics Score Formula 4](#_Toc15606)

[SUPPLEMENTARY FIGURES 5](#_Toc21419)

[Supplementary Fig. 1. 5](#_Toc10654)

[Supplementary Fig. 2. . 6](#_Toc11962)

[Supplementary Fig. 3. 7](#_Toc19191)

[SUPPLEMENTARY TABLES 8](#_Toc29250)

[Table S1. . 8](#_Toc19534)

[Table S2. 10](#_Toc17036)

[Table S3 11](#_Toc13852)

.

# SUPPLEMENTARY METHODS

## Radiomics Procedure

All patients underwent standard pretreatment adrenal MRI with 3.0T MR scanners (Intera Achieva, Philips Medical Systems, Best, the Netherlands; or Siemens Magnetom Skyra, Germany). Axial T2-weighted Digital Imaging and Communications in Medicine (DICOM) images were retrieved for radiomics analysis. And the T2-weighted image acquisition parameters were as follows: slice thickness, 3 mm; repetition time, 2500-3500 msec; echo time, 80-120 msec (Sun Yat-sen Memorial Hospital of Sun Yat-sen University); slice thickness, 5 mm; repetition time, 4425 msec; echo time, 88 msec (Third Affiliated Hospital of Sun Yat-sen University).

MRI images were reviewed by a radiologist with 10 years of experience in radiology and then verified by an experienced radiologist with 19 years of experience in radiology. Discrepancies between the two radiologists were resolved by consensus. As for the imaging diagnosis of pheochromocytoma, the interclass correlation coefficient was 0.871 in our study. Radiologists were blinded to the patients’ clinical information. Volumes of interest (VOIs) of the adrenal lesions were semi-automatically segmented using the *GrowCut* segmentation method implemented in the publicly available 3D Slicer software (1). *GrowCut* is an interactive region-growing segmentation method (2). Using the *GrowCut* method, the VOI is initially delineated. Then, radiologists can meticulously edit the boundary of the regions of interest (ROIs) slice-by-slice by erasing or drawing the mask manually to improve the alignment of the ROIs with the tumor outlines. Since the boundary between a tumor and nearby normal soft tissues is not always well defined, a conservative approach to contouring was taken to remain within the tumor, even at the risk of not including a small part of tumor edges. As a result, such VOI was defined for each patient for radiomics feature extraction.

In this study, 1301 radiomics features were extracted from the defined VOI using the *PyRadiomics* platform implanted in 3D Slicer software (1). As part of image preprocessing, images were resampled to isotropic voxels with 1-mm sides using a B Spline interpolator (1,3). Target region intensity values were discretized using a bin width of 25. Aside from the original image, features were also extracted from wavelet and Laplacian of Gaussian (LoG)-filtered images. For the wavelet filter, each image was filtered using either a high-bandpass filter or a low-bandpass filter in the x, y and z directions, yielding 8 different combinations of decompositions. For the LoG filter, images were filtered using a 3D LoG filter implemented in SimpleITK and by changing sigma values to 5.0, 4.0, 3.0, 2.0 and 1.0 mm, yielding another 5 derived images.

The features were divided into seven categories: (a) first-order statistics, (b) shape-based, (c) Gray Level Cooccurence Matrix (GLCM), (d) Gray Level Run Length Matrix (GLRLM), (e) Gray Level Size Zone Matrix (GLSZM), (f) Neighboring Gray Tone Difference Matrix (NGTDM), and (g) Gray Level Dependence Matrix (GLDM). We extracted 18 first-order features, 23 GLCM features, 16 GLRLM features, 16 GLSZM features, 5 NGTDM features and 14 GLDM features from each original (unfiltered) image as well as filtered images. Combined with the 13 shape features extracted from the unfiltered image, this process yielded a total of 1301 features extracted from each VOI. Detailed information about the feature names and mathematical formulas can be obtained from the *PyRadiomics* documentation available at http://pyradiomics.readthedocs.io/en/latest.

To assess the feature extraction reproducibility, interclass correlation coefficients (ICCs) were used to assess the interobserver reproducibility of the radiomics feature extraction. The ICC is a statistical measure, ranging between 0 and 1, indicating null and perfect reproducibility, respectively. An ICC greater than 0.75 represents good agreement (4). We initially chose 40 random MRI images (with 40 adrenal lesions) for VOI segmentation and feature extraction. The VOI segmentation was performed in a blind fashion by two radiologists, i.e., reader 1 and reader 2. As a result, satisfactory interobserver feature extraction reproducibility was achieved (ICC, mean ± SD, 0.762 ± 0.294). Therefore, all outcomes were based on the features extracted by reader 1.

## Radiomics Score Formula

Radiomics score =

-5.133048671

-0.001657734 × LoG (σ=3)_First order_10 Percentile

-0.0000256 × LoG (σ=3)_First order_Mean

-0.008656211 × LoG (σ=4)_First order_Mean

-0.040735064 × Wavelet (LLL)_First order_Skewness

+0.169785339 × GLDM_Dependence Entropy

+3.010282787 × GLCM_Correlation

+0.001789298 × First order_Interquartile Range

# SUPPLEMENTARY FIGURES


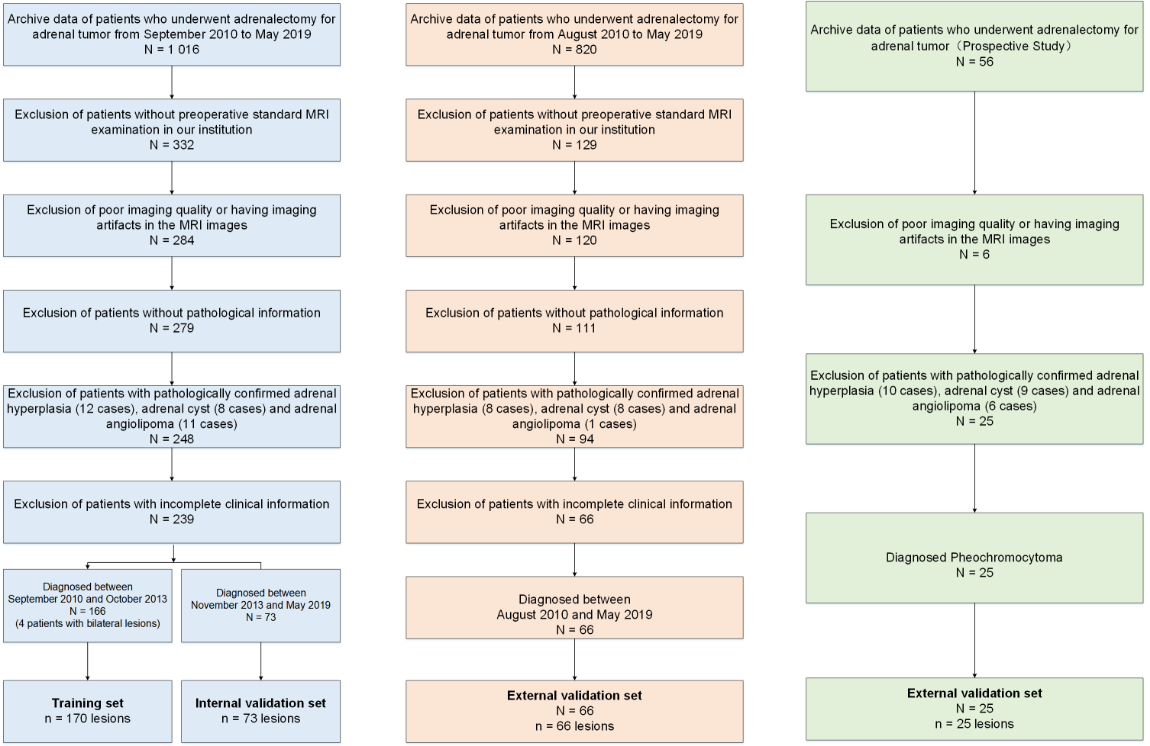


## Supplementary Fig. 1. Recruitment pathways.


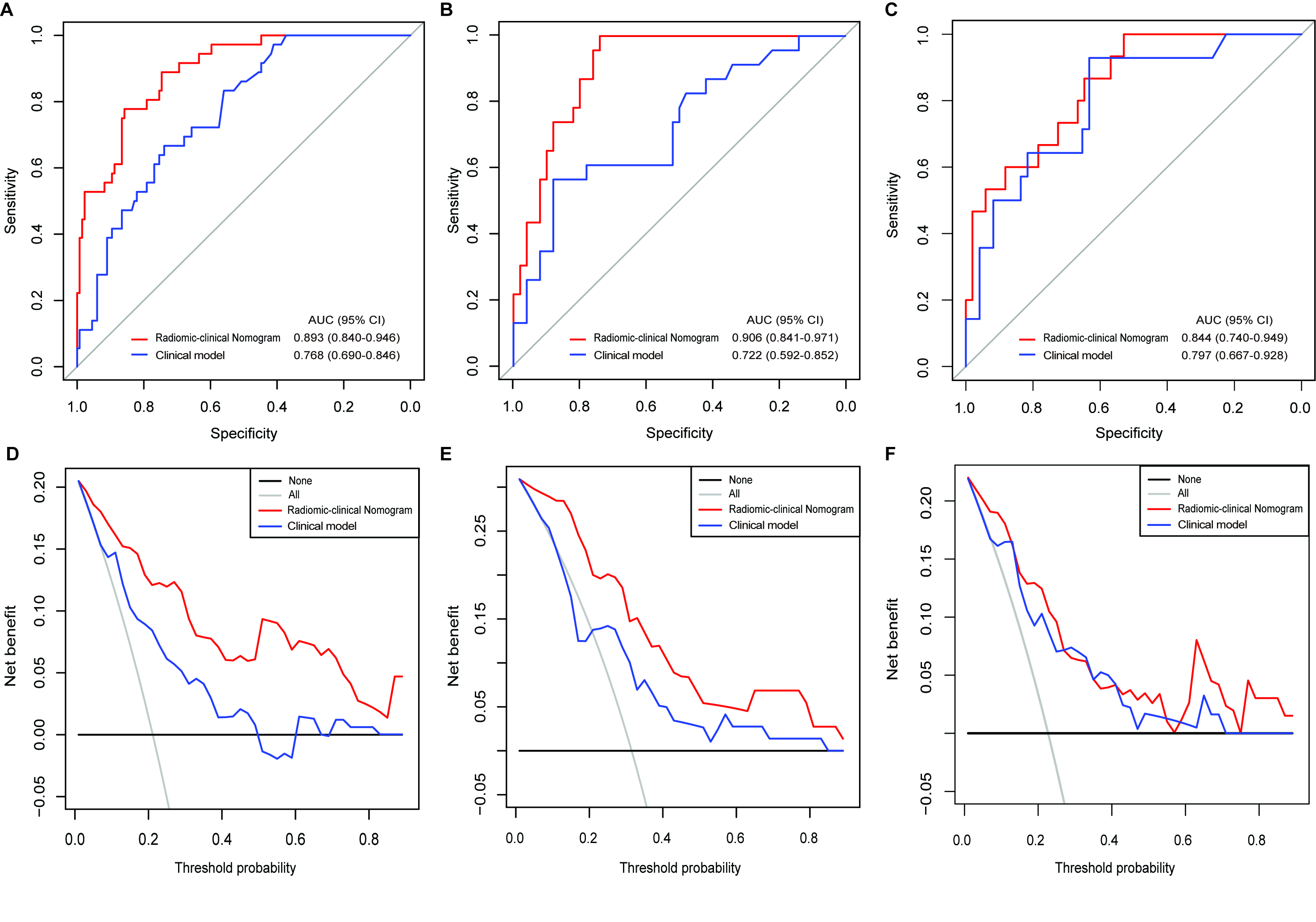


## **Supplementary Fig. 2.** Receiver operating characteristic analysis (A-C) and decision curve analysis (D-F) of radiomic-clinical nomogram model and clinical model in the training set, internal validation set, and external validation set, respectively.


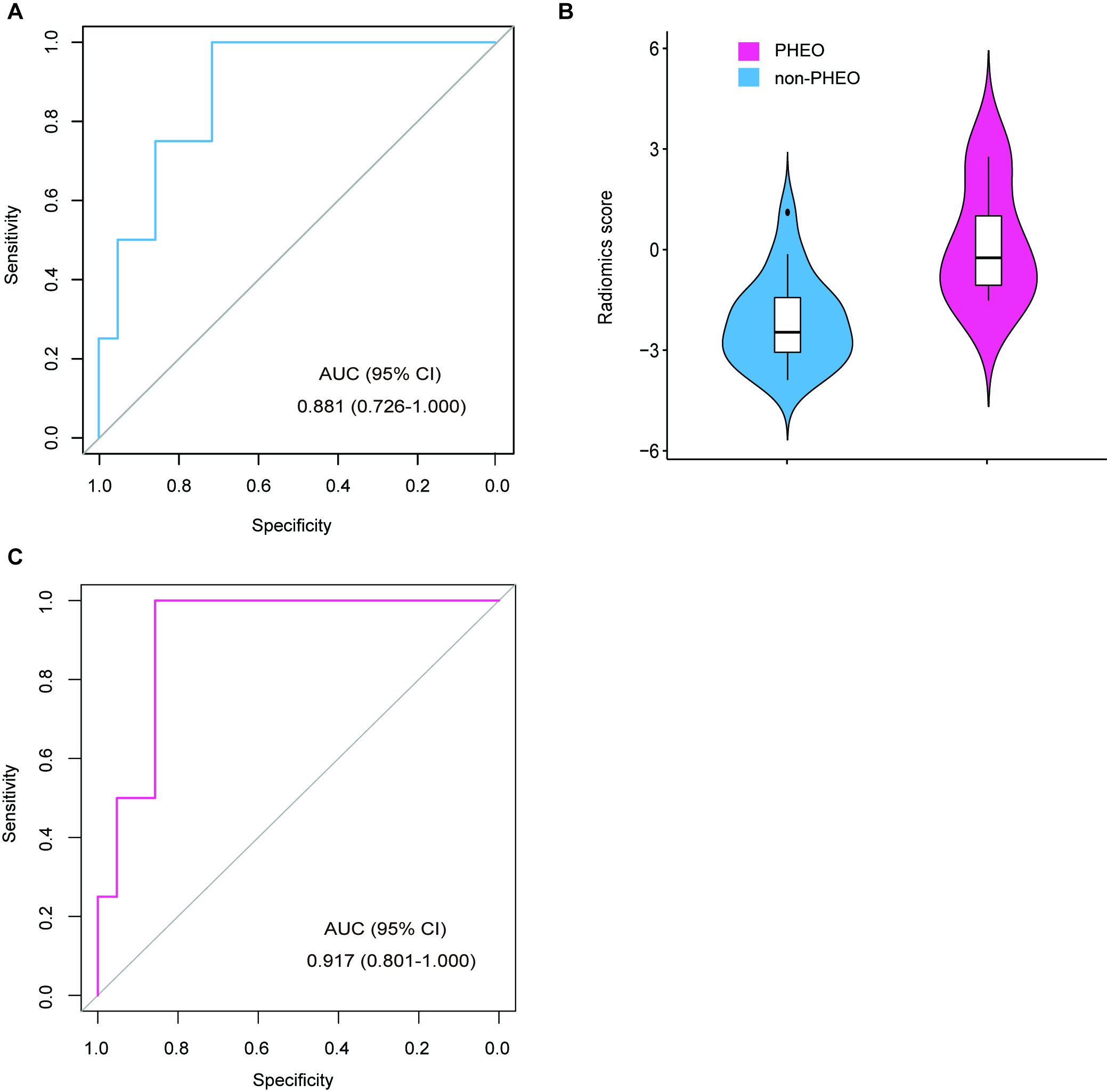


## **Supplementary Fig. 3.** Performance of the radiomics signature and radiomic-clinical model in the prospective validation set. (A) ROC curve of the radiomics signature in the prospective validation set. (B) Boxplots of the radiomics score in the prospective validation set. (C) ROC curve of the radiomic-clinical nomogram in the prospective validation set

# SUPPLEMENTARY TABLES

## Table S1. The clinical data of the prospective validation set.

| ID | Age (years) | Sex | Smoke | Drunk | Symptom number^*^ | Headache | Palpitation | Diaphoresis | Hypertension | Tumor size (cm) | Tumor location | MRI_PHEO^**^ | Path_PHEO^***^ |
| --- | --- | --- | --- | --- | --- | --- | --- | --- | --- | --- | --- | --- | --- |
| Patient1 | 53 | Male | Yes | No | 0 | No | No | No | No | 4.58 | Right | Yes | Yes |
| Patient2 | 50 | Female | No | No | 1 | No | Yes | No | Yes | 2.8 | Left | No | No |
| Patient3 | 59 | Female | No | No | 0 | No | No | No | Yes | 1.2 | Left | No | No |
| Patient4 | 60 | Female | No | No | 0 | No | No | No | No | 3.4 | Left | No | No |
| Patient5 | 64 | Female | No | No | 0 | No | No | No | Yes | 2.25 | Left | No | No |
| Patient6 | 8 | Male | No | No | 0 | No | No | No | No | 3 | Right | No | No |
| Patient7 | 56 | Female | No | No | 1 | Yes | No | No | Yes | 6.7 | Right | Yes | Yes |
| Patient8 | 47 | Male | No | No | 0 | No | No | No | Yes | 3.2 | Left | No | No |
| Patient9 | 59 | Male | No | No | 0 | No | No | No | Yes | 1 | Left | No | No |
| Patient10 | 43 | Female | No | No | 1 | Yes | No | No | Yes | 1.3 | Left | No | No |
| Patient11 | 43 | Female | No | No | 0 | No | No | No | Yes | 1.7 | Right | No | No |
| Patient12 | 40 | Male | Yes | Yes | 0 | No | No | No | Yes | 1.3 | Left | No | No |
| Patient13 | 64 | Male | Yes | Yes | 0 | No | No | No | No | 4.2 | Left | No | No |
| Patient14 | 46 | Female | No | No | 1 | Yes | No | No | No | 4.99 | Right | No | No |
| Patient15 | 41 | Male | No | No | 1 | Yes | No | No | No | 2.4 | Left | No | No |
| Patient16 | 34 | Female | No | No | 0 | No | No | No | No | 9.8 | Left | No | No |
| Patient17 | 56 | Male | No | No | 0 | No | No | No | Yes | 2.8 | Right | No | No |
| Patient18 | 52 | Female | No | No | 0 | No | No | No | Yes | 11.7 | Left | No | No |
| Patient19 | 25 | Male | No | No | 1 | No | No | Yes | Yes | 1.6 | Left | No | No |
| Patient20 | 49 | Male | Yes | No | 0 | No | No | No | Yes | 2.4 | Right | No | No |
| Patient21 | 53 | Female | No | No | 0 | No | No | No | No | 4.5 | Left | No | No |
| Patient22 | 60 | Male | No | No | 3 | No | Yes | Yes | Yes | 3.4 | Right | Yes | Yes |
| Patient23 | 63 | Male | No | No | 0 | No | No | No | Yes | 1 | Right | No | No |
| Patient24 | 73 | Female | No | No | 0 | No | No | No | Yes | 6.3 | Left | Yes | Yes |
| Patient25 | 55 | Male | No | No | 0 | No | No | No | Yes | 1.8 | Right | No | No |

^*^ Symptoms include headache, palpitation, and diaphoresis.

^**^MRI_PHEO means that the diagnosis of pheochromocytoma was based on MRI.

^***^Path_PHEO denotes pathological diagnosis of pheochromocytoma.

## Table S2. Stratified analysis of the association between the radiomics signature and PHEO in the combined training, internal validation and external validation sets.

|  |  | **Radiomics score** | |  |
| --- | --- | --- | --- | --- |
|  | **Subgroups** | **non-PHEO** | **PHEO** | ***P*** |
| **Sex** | Male | -1.918 (-2.386 to -1.135) | -0.824 (-1.135 to -0.079) | < 0.001 |
|  | Female | -1.945 (-2.376 to -1.217) | -0.685 (-1.055 to -0.215) | < 0.001 |
| **Age, years** | < 65 | -1.949 (-2.381 to -1.183) | -0.657 (-1.077 to -0.151) | < 0.001 |
|  | ≥ 65 | -1.694 (-2.416 to -0.983) | -1.054 (-1.213 to -0.808) | 0.044 |
| **Tumor location** | Left | -2.201 (-2.634 to -1.460) | -0.746 (-1.064 to -0.372) | < 0.001 |
|  | Right | -1.756 (-2.209 to -1.084) | -0.762 (-1.099 to 0.039) | < 0.001 |
| **MRI-determined tumor size** | < 3.3 cm | -2.222 (-2.612 to -1.859) | -0.973 (-1.130 to -0.206) | < 0.001 |
|  | ≥ 3.3 cm | -1.168 (-1.698 to -0.830) | -0.720 (-1.069 to -0.143) | < 0.001 |
| **Smoker** | Yes | -2.054 (-2.453 to -1.044) | -0.778 (-0.922 to 0.022) | < 0.001 |
|  | No | -1.915 (-2.372 to -1.183) | -0.727 (-1.096 to -0.196) | < 0.001 |
| **Hypertension** | Yes | -2.108 (-2.409 to -1.455) | -0.564 (-1.123 to -0.028) | < 0.001 |
|  | No | -1.600 (-2.266 to -1.016) | -0.780 (-1.073 to -0.490) | < 0.001 |
| **Slice thickness** | 3 mm | -1.906 (-2.386 to -1.119) | -0.818 (-1.099 to -0.153) | < 0.001 |
|  | 5 mm | -1.983 (-2.396 to -1.285) | -0.619 (-1.080 to -0.131) | < 0.001 |
